# The association of number and geographic proximity of children with care home use before all-cause and dementia deaths: a register-based study of Finnish older adults

**DOI:** 10.1093/geronb/gbaf250

**Published:** 2025-12-10

**Authors:** Kaarina Korhonen, Elina Einiö, Pekka Martikainen

**Affiliations:** Helsinki Institute for Demography and Population Health, Faculty of Social Sciences, University of Helsinki, Helsinki, Finland; Max Planck—University of Helsinki Center for Social Inequalities in Population Health, Faculty of Social Sciences, University of Helsinki, Helsinki, Finland; Helsinki Institute for Demography and Population Health, Faculty of Social Sciences, University of Helsinki, Helsinki, Finland; Max Planck—University of Helsinki Center for Social Inequalities in Population Health, Faculty of Social Sciences, University of Helsinki, Helsinki, Finland; Helsinki Institute for Demography and Population Health, Faculty of Social Sciences, University of Helsinki, Helsinki, Finland; Max Planck—University of Helsinki Center for Social Inequalities in Population Health, Faculty of Social Sciences, University of Helsinki, Helsinki, Finland; (Social Sciences Section)

**Keywords:** Nursing home, Intergenerational caregiving, Family structure, Socioeconomic status, Proximity to death

## Abstract

**Objectives:**

Demographic trends, including smaller and more geographically dispersed families, may limit the availability of family caregiving as an alternative to care home residence. This study examined how care home residence varies according to the number and geographic proximity of adult children.

**Methods:**

Using Finnish register data on cohorts born in 1938–1953, we examined care home days during the 5 years preceding death at age ≥65, or up to December 31, 2018 for survivors. Poisson regression models estimated incidence rate ratios for annual care home days by number of children (nationwide cohort, *n = *1,058,184) and geographic proximity of children based on household and ZIP codes (Helsinki metropolitan sample, *n = *136,674). To account for health and care needs by design, we conducted separate analyses for the full cohort, decedents, and those who died from dementia-related causes.

**Results:**

Compared to parents with two children, childless women and men spent more days in care homes, while the number of children among parents did not matter when adjusting for marital and socioeconomic status and accounting for proximity to death (deceased subsample). For men without spouses, having children was particularly important. Shorter distance to children was associated with fewer care home days among women. Among individuals who died from dementia, having children was not associated with care home use, and associations with geographic proximity were weak.

**Discussion:**

Adult children may substitute for care homes in the general population, but their impact appears small in cases of severe cognitive impairment such as dementia.

As populations age and long-term care costs rise, many countries increasingly rely on family caregiving to help older adults live at home (Ranci & Pavolini, 2015; van den Broek et al., 2019). However, demographic trends—such as smaller family sizes, declining intergenerational co-residence, and greater geographic dispersion of families—raise concerns about the availability of adult children to reduce care home admissions (Chudnovskaya & Kolk, 2017; Ruggles, 2007; Spijker et al., 2022). Yet, it is uncertain whether having more children or living close to them reduces care home use, especially among older adults with dementia, who represent a substantial share of care home residents (Finne-Soveri et al., 2014; Harris-Kojetin et al., 2016).

A large body of research shows that older adults with children are less likely than those who are childless to enter care homes in later life (Arber & Ginn, 1993; Aykan, 2003; Désesquelles et al., 2003; Freedman, 1996; Gaugler et al., 2007; Grundy & Jitlal, 2007; Hedinger et al., 2015; Syse et al., 2022). Possibly reflecting greater caregiving availability (Grundy & Read, 2012; Tomassini et al., 2004), a higher number of children has also been linked to a lower likelihood of care home entry (Aykan, 2003; Coward et al., 1996; Gaugler et al., 2007; Himes et al., 2000; Syse et al., 2022). However, most studies assume a linear relationship, overlooking potential diminishing returns—or even negative effects—associated with having many children. While studies on parental health and mortality suggest that parents with only one child or four or more children tend to experience poorer health and lower survival in later life compared to those with two or three children (Barclay et al., 2016; Einiö et al., 2016), few studies explicitly test for non-linearity for care home residence. Most research also focuses exclusively on women (Grundy & Jitlal, 2007), leaving the impact among older men underexplored.

Intergenerational co-residence has been linked to a lower likelihood of care home entry (Artamonova et al., 2023; Grundy & Jitlal, 2007; van der Pers et al., 2015). Among non-co-residing families, geographic proximity to children is associated with a greater likelihood of receiving support, as a closer distance facilitates more frequent contact and assistance (Bordone, 2009; Hank, 2007; Mulder & van der Meer, 2009). However, evidence on whether this increased caregiving reduces care home use remains limited. A Dutch study found that living in the same neighborhood as one’s offspring reduced the likelihood of care home entry compared to living within 5 km (van der Pers et al., 2015). A Swedish study reported similar findings when comparing living in the same neighborhood to more than 20 km away; although for men, this effect was only observed when the nearest child was a son (Artamonova et al., 2023). Both longitudinal studies accounted for health by adjusting for death occurring within 1–2 years. Since older adults already residing in care homes at baseline were excluded, this approach might have omitted individuals who died from causes requiring extended care, particularly dementia. Further research is needed to assess the impact of geographic proximity on care home residence, with a more comprehensive consideration of health, in particular, cognitive disability.

Children may play an especially important role when parents’ health deteriorates (Artamonova et al., 2023; Grundy & Read, 2012). However, when care needs become substantial—as in advanced dementia—the ability of children to substitute for formal care may be limited (Bonsang, 2009). Prior evidence suggests distinct patterns for the effects of marriage on care home use: while having a spouse becomes increasingly important towards the end of life in the general older population, its influence diminishes in the later stages of dementia (Korhonen et al., 2018). The cognitive decline, functional limitations, and behavioral symptoms characteristic of dementia can place substantial demands on family caregivers. Because dementia is a major predictor of care home entry, it is crucial to assess whether the number of children and their geographic proximity relate to care home residence in this population. To our knowledge, no prior study has done so.

## Aims of the study

This study analyzes how care home residence varies according to the number and geographic proximity of adult children, using Finnish population register data that include nearly complete fertility histories for the full cohort of men and women born between 1938 and 1953. Care home residence is measured as the annual number of days in care over a 5-year period, based on national care registers.

We use two analytic study populations (nationwide cohort and Helsinki metropolitan sample) and three model specifications that reflect differences in health status and dementia-related care needs. First, we assess the association between the number of children and care home residence in the nationwide cohort, using a categorical variable to capture potential non-linear effects. We also test whether the importance of children differs between those with and without spouses, given that spouses often provide care (Arber & Ginn, 1991; Huber et al., 2009). Second, we analyze how care home residence varies by the geographic proximity of the nearest child among parents living in the Helsinki metropolitan area. Information on ZIP codes, which in this region consist of relatively small areas (median 2.7 km^2^), allows distinguishing between close and longer distances more accurately (children could live anywhere in Finland).

To account for health status and cognitive impairment, we conduct all analyses separately for (1) the full cohort, (2) cohort members who died at age ≥65 years (in 2003–2018), and (3) those who died from dementia-related causes. This design uses proximity to death and cause of death as indicators of care needs, mitigating potential overestimation of the effects associated with children. Unlike time-to-event analyses, this approach focuses on cumulative time spent in care homes rather than the age at entry, which can be influenced by differences in survival. We also present models adjusted for marital and socioeconomic status, which may confound intergenerational associations in care home residence.

## The Finnish context

Finland represents a Nordic welfare regime traditionally characterized by a “public service model,” in which the state has assumed primary responsibility for elder care through universally accessible, needs-based long-term care services (Anttonen & Sipilä, 1996). Compared to many other countries, families have played a relatively limited role in providing high-intensity care (Rostgaard et al., 2022). However, since the 1990s, cost-saving pressures have led to a gradual retrenchment in public long-term care provision across the Nordic countries, including Finland (Ranci & Pavolini, 2015; Rostgaard et al., 2022). Care homes and formal in-home care services are increasingly targeted at individuals with the most severe care needs—typically those with dementia—and the transition to care homes has been delayed to the later stages of life (Forma et al., 2017; Korhonen et al., 2024). This shift has been accompanied by growing emphasis on informal family caregiving, supported by financial incentives (Rostgaard et al., 2022). As a result, care services in Finland remain publicly provided and needs-assessed, but emerging gaps in formal care may increase reliance on informal support, particularly from adult children.

## Methods

### Data and study population

We leveraged data from Statistics Finland’s population register, which covers all Finnish residents and links information of individuals via personal identification numbers to other administrative registers. For the purposes of this study, we restricted the study population to cohorts born between 1938 and 1953 (*n = *1,058,184), for whom the linkage of biological and adopted children was near perfect (Einiö et al., 2016). We formed two study samples, one including all Finnish residents born in the given years, and one restricted to those living in the Helsinki metropolitan area. Both samples were further broken down into those who died between 2003 and 2018 and those who died from dementia-related causes. For the deceased, we obtained the exact date of death from the Cause of Death Register maintained by Statistics Finland. We identified individuals who died from dementia-related causes using the International Classification of Diseases 10th Revision, with the underlying or contributing cause of death coded as F01 (vascular dementia), F03 (unspecified dementia), G30 (Alzheimer’s disease), and R54 (senility).

The Finnish Institute for Health and Welfare maintains a national register of health and social care services covering both public and private providers. Care home residences included nursing homes, service housing with 24-hr assistance, health centers, hospitals, and rehabilitation facilities when stays lasted at least 90 days or were based on an administrative long-term care decision. The 90-day criterion allowed for short transfers between sites, with no more than one night between residence periods. Exit dates were missing for 4% of stays; in these cases, we assumed the stay ended the day after the last end-of-year patient census, confirming the person was still in care.

We extracted data on care home stays during the final 5 years of observation: 2014–2018 for those alive at the end of 2018, and the 5 years preceding death for those who died aged ≥65 years between 2003 and 2018 (see Figure S1). The number of care home days was calculated annually over this period (5 × 365 days). Individuals who emigrated were excluded, as non-residents are ineligible for long-term care in Finland (*n = *9,528).

The study was approved by the Statistics Finland Board of Ethics and the Social and Health Data Permit Authority Findata (permit numbers TK/3343/07.03.00/2023 and THL/499/14.06.00/2024). Statistics Finland pseudonymized the data prior to providing it to researchers.

### Number and geographic proximity of children

In Finland, each child has a personal identification code that links them to their biological or adoptive parents. Parent-child links were obtained from Statistics Finland. The number of living children each year was categorized as no children, 1, 2, 3, or ≥4 children.

Geographic proximity was determined from household identifiers and residential ZIP codes of parents and children. Because ZIP code areas vary greatly in size across Finland (i.e., being smaller and more precise in urban regions), we restricted proximity analyses to parents living in the Helsinki metropolitan area (Espoo, Helsinki, Vantaa, and Kauniainen), which includes 168 ZIP codes with a median area of 2.7 km^2^. Children could live anywhere in Finland. Distances between ZIP code centroids were provided by Statistics Finland. Co-residence was identified via household ID.

Proximity was defined based on the nearest living child at the start of the 5-year observation period (ie, the end of the preceding year) and only for parents not in care homes at that time, to reflect their situation before potential entry. Geographic proximity was categorized as co-resident, same ZIP code (not co-resident), <10 km, 10 to <50 km, and ≥50 km, and unknown or child lives abroad.

### Sociodemographic characteristics

Gender (women, men) was based on the most recently confirmed gender in the population register. Age was measured at death or at the end of 2018 for survivors. Region of residence was included to account for regional variation in family formation patterns and health policies and health status in the analysis for the nationwide analyses. It was classified according to Eurostat’s Nomenclature des Unités Territoriales Statistiques level 3, corresponding to 19 regions in Finland.

Marital status (married, widowed, divorced, never married) was used as a time-varying covariate to account for changes during the observation period, such as bereavement. Individuals cohabiting but not married were classified by their legal marital status, as our focus was on marital rather than cohabitation status to avoid overlap with care home residence.

Education level was classified according to the highest attained degree: tertiary (International Standard Classification of Education [ISCED]-2011 5–8), secondary (ISCED 3–4), and no qualifications (ISCED 0–2). Inflation-adjusted disposable income was calculated as a 3-year average preceding the observation period, including the person’s earned, entrepreneurial, and property income, as well as transfers received, but excluding taxes and tax-deductible expenses. Income was divided into quintiles within 5-year age groups to account for age-related differences in earnings.

### Statistical analyses

We estimated incidence rate ratios (IRR) for annual care home days by the number of children using population-averaged Poisson regression models for panel data with robust standard errors. To account for within-individual correlation, we applied an autoregressive correlation structure (ar1), assuming stronger correlation between observations closer in time (Twisk, 2003).

To evaluate the contribution of demographic and socioeconomic factors, we fitted three adjustment models. Model 1 adjusted for age, age squared, region, and calendar year, model 2 additionally for time-varying marital status, and model 3 further for education and income. Separate models were estimated for the full cohort, all deceased, and those who died from dementia-related causes. The full-cohort analysis followed a conventional prospective design without health adjustments, whereas for the deceased, the observation period was anchored to the date of death, effectively controlling for proximity to death by design.

We next analyzed whether associations between the number of children and care home use differed by marital status. In the interaction analyses, we dichotomized marital status into married versus non-married but allowed the status to change over time. IRRs are presented from stratified models adjusted as in model 3. Overall interaction as well as individual interaction terms were formally tested using the Wald *χ*^2^ test.

Finally, we analyzed IRRs for care home days by geographic proximity to the nearest child among parents in the Helsinki metropolitan area who were not in care homes at the start of the observation period. These models also adjusted for the number of children, since closer proximity is more likely for parents with more children (Holmlund et al., 2013). Region of residence was excluded from covariates. Stata 18.0 was used in all estimation (StataCorp., 2023).

### Sensitivity and supplementary analyses

Because the annual number of care home days showed a bimodal distribution, with most individuals having either no days or full-year residence (Supplementary Table 1, see online supplementary material), we additionally estimated logistic regression models for any care home use. This approach helps assess potential violations of Poisson model assumptions arising from excess zeros and concentration at the upper limit.

Since previous studies suggest that the presence of daughters, compared to sons, plays a more crucial role (Freedman, 1996; Himes et al., 2000), we conducted supplementary analyses to examine whether having at least one daughter or having a daughter as the nearest child is associated with care home residence.

## Results

Table 1 presents the characteristics of the full nationwide cohort, all deceased, and those who died from dementia-related causes. In the full cohort, the average number of annual care home days was 6.4 (*SD* 45.7) among women and 6.7 (*SD* 46.6) among men. Among all deceased, women spent a yearly average of 34.3 days (*SD* 101.0) and men 23.5 days (*SD* 84.4) in care homes during the last 5 years of their lives. Individuals who died from dementia-related causes had substantially more care home days, with women averaging 154.2 days (*SD* 171.0) and men 111.9 days (*SD* 158.1).

**Table 1.** Distribution of person-years in the full cohort, among all deceased, and among those who died from dementia-related causes, a nationwide cohort of Finnish women and men, 1998–2018.

Characteristics	Women			Men		
	Full cohort	Deceased		Full cohort	Deceased	
		All causes of death	Dementia		All causes of death	Dementia
**Annual care home days, Mean (*SD*)**	6.4 (45.7)	34.3 (101.0)	154.2 (171.0)	6.7 (46.6)	23.5 (84.4)	111.9 (158.1)
**Age in years^a^, Mean (*SD*)**	71.5 (4.4)	70.6 (4.2)	73.5 (4.0)	71.1 (4.3)	70.0 (4.0)	73.1 (4.1)
**Number of children, %**						
** No children**	14.4	18.8	17.5	18.2	24.2	22.4
** 1 child**	21.6	22.4	21.0	19.2	19.8	18.9
** 2 children**	39.1	33.6	34.3	37.6	32.9	33.6
** 3 children**	17.5	16.6	17.6	17.3	15.5	16.5
** ≥4 children**	7.4	8.7	9.6	7.7	7.6	8.5
**Marital status, %**						
** Married**	54.1	45.6	46.0	65.0	53.4	58.6
** Widowed**	15.9	18.4	23.3	4.6	5.4	6.9
** Divorced**	20.2	23.2	19.6	17.2	23.0	19.3
** Never married**	9.8	12.8	11.1	13.1	18.2	15.3
**Education, %**						
** High**	26.2	17.0	16.5	27.1	18.1	18.6
** Intermediate**	35.0	29.8	29.0	33.1	28.8	26.7
** Low**	38.8	53.2	54.6	39.8	53.1	54.7
**Income, %**						
** Highest quintile**	13.1	6.8	7.0	27.4	14.6	15.5
** 4th**	18.7	11.4	12.4	21.4	15.3	18.0
** 3rd**	21.4	17.3	17.9	18.5	18.4	21.9
** 2nd**	22.4	26.0	27.9	17.3	23.7	23.7
** Lowest quintile**	24.4	38.5	34.9	15.4	28.0	20.9
**Person-year**	2,729,065	223,540	19,550	2,561,855	389,820	21,205
***N* of individuals**	545,813	44,708	3910	512,371	77,964	4241

a Age at the end of 2018, or age at death for the deceased.

In the full cohort, 14.4% of women and 18.2% of men had no children, while about two in five (39.1% of women and 37.6% of men) had two children. Women were less likely to be married than men. Among all deceased individuals and those who died from dementia-related causes, a larger proportion were childless compared to the full cohort. The deceased were also more likely to be widowed and had lower levels of education and income.

Table 2 presents the distribution of person-years among parents living in the Helsinki metropolitan area. At the start of the observation period, 9.5% of all mothers and 12.7% of all fathers co-resided with their child 16.1% of mothers and 13.5% of fathers lived in the same ZIP code as their nearest child but did not co-reside, and nearly half lived within 10 km (49.9% of mothers and 48.0% of fathers). Parents in the metropolitan population tended to have higher education and income levels compared to the full nationwide cohort (Table 1).

**Table 2.** Distribution of person-years in the full cohort, among all deceased, and among those who died from dementia-related causes, Finnish women and men with at least one child and living in the Helsinki metropolitan area, 1998–2018.

Characteristics	Women			Men		
	Full cohort	Deceased		Full cohort	Deceased	
		All causes of death	Dementia		All causes of death	Dementia
**Annual care home days, Mean (*SD*)**	3.1 (31.2)	29.4 (91.9)	114.6 (157.1)	3.1 (30.6)	13.0 (61.3)	93.8 (147.3)
**Age in years,^a^ Mean (*SD*)**	71.5 (4.3)	74.9 (6.1)	79.7 (5.0)	71.2 (4.3)	69.9 (4.0)	73.4 (3.9)
**Geographic proximity, %**						
** Co-resident**	9.5	8.9	7.5	12.7	11.4	7.6
** Same ZIP code**	16.1	16.3	17.1	13.5	12.2	15.4
** <10 km**	49.9	50.1	51.5	48.0	46.6	51.9
** 10–<50 km**	18.8	19.1	18.9	19.4	21.5	16.9
** ≥50 km**	4.0	4.1	3.4	5.0	6.8	6.3
** Unknown or child abroad**	1.6	1.5	1.5	1.4	1.6	2.0
**Number of children, %**						
** 1 child**	32.4	32.5	30.9	27.3	31.0	31.1
** 2 children**	47.3	41.3	41.8	47.4	44.7	44.8
** 3 children**	15.8	18.3	19.0	18.2	17.3	16.9
** ≥4 children**	4.6	7.8	8.2	7.1	7.0	7.2
**Marital status, %**						
** Married**	53.4	40.6	39.5	71.9	60.4	68.2
** Widowed**	14.3	27.4	34.8	4.7	5.8	6.0
** Divorced**	28.2	28.9	23.3	21.2	31.9	24.5
** Never married**	4.1	3.1	2.4	2.2	1.9	1.3
**Education, %**						
** High**	38.0	20.5	21.1	45.6	31.3	36.0
** Intermediate**	27.6	21.0	19.0	25.5	25.7	22.9
** Low**	34.4	58.5	60.0	28.8	43.0	41.1
**Income, %**						
** Highest quintile**	25.7	13.8	15.5	48.8	27.2	32.7
** 4th**	24.2	16.3	19.0	20.6	19.3	21.2
** 3rd**	21.5	22.4	20.8	13.2	18.5	23.7
** 2nd**	16.6	25.2	23.1	9.7	18.9	12.6
** Lowest quintile**	12.1	22.3	21.6	7.7	16.1	9.8
**Person-years**	373,110	29,585	1945	309,799	43,613	1985
***N* of individuals**	74,683	5925	390	61,991	8729	397

a Age at the end of 2018, or age at death for the deceased.

## Number of children

**Figure 1.**
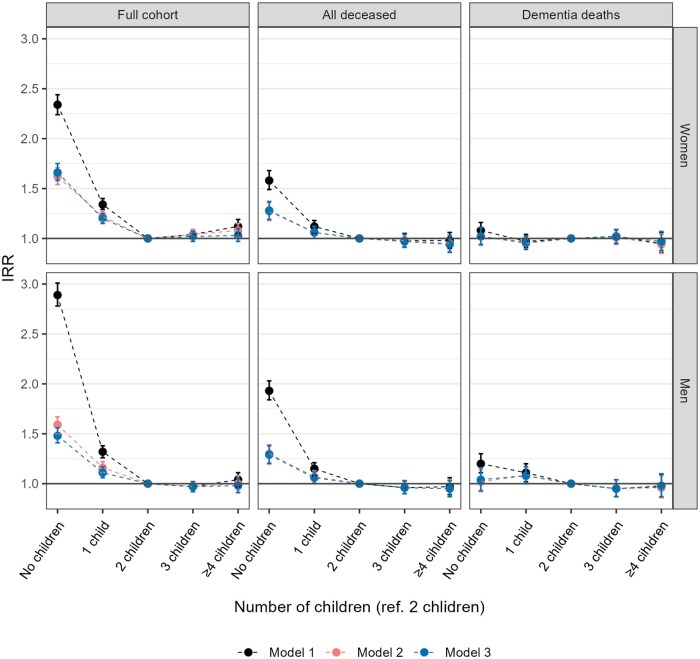
Incidence rate ratios with their 95% CI for annual care home days by the number of children in the full cohort, among all deceased, and those who died from dementia-related causes, a nationwide cohort of Finnish women and men, 1998–2018. *Note*. Model 1 adjusted for age, age squared, region, and calendar year; Model 2 adjusted for covariates in model 1 + marital status; Model 3 adjusted for covariates in model 2 + education level and income.

Figure 1 presents the IRRs for annual care home days by the number of children (see Supplementary Table 2, see online supplementary material, for exact estimates). For women in the full cohort, IRRs in model 1 followed an inverse J-shaped curve, with those having no children (IRR = 2.34, 95% CI: 2.24–2.44), one child (IRR = 1.34, 95% CI: 1.29–1.40) or four or more children (IRR = 1.12, 95% CI: 1.05–1.19) spending more days in care homes compared to those with two children. Among men, IRRs for care home days were higher for those without children (IRR = 2.89, 95% CI: 2.78–3.01) and with one child (IRR = 1.32, 95% CI: 1.26–1.38) compared to men with two children. Adjusted for marital status in model 2, these differences were greatly attenuated. In model 3, further adjustment for education and income attenuated the elevated IRR, especially among men without children. However, women and men without children (women: IRR = 1.66, 95% CI: 1.58–1.75; men: IRR = 1.48, 95% CI: 1.41–1.56) or with only one child (women: IRR = 1.20, 95% CI: 1.15–1.25; men: IRR = 1.11, 95% CI: 1.06–1.17) had a higher incidence of care home days compared to parents of two children.

The analysis for deceased individuals showed considerably smaller relative differences in care home days by the number of children, suggesting important health-based selection. Adjusted for marital status in model 2, care home residence among women and men with one child did not differ from those with two children, while IRRs for those without children remained elevated. Adjustment for socioeconomic status in model 3 had nearly no impact on the estimates (women: IRR = 1.28, 95% CI: 1.19–1.37; men: IRR = 1.29, 95% CI: 1.20–1.38). For individuals who died from dementia-related causes, the number of children was not associated with care home days when adjusted for marital status (model 2) and socioeconomic status (model 3).

The association between having children and care home residence was more pronounced among men without a spouse, compared to those who were married. This pattern was observed both in the full cohort and among deceased men, but not among men who died from dementia-related causes (Table 3). Among women without spouses, having four or more children tended to be associated with a lower incidence of care home days, yet this was statistically significant only among all deceased women (IRR = 0.84; 95% CI: 0.76–0.94).

**Table 3.** Incidence rate ratios with their 95% *CI* for annual care home days by the number of children and marital status in the full cohort, among all deceased, and those who died from dementia-related causes, a nationwide cohort of Finnish women and men, 1998–2018.

Variable	Women				Test for interaction		Men					Test for interaction	
	Married		Non-married				Married			Non-married			
	IRR	95% CI	IRR	95% CI	*χ* ^2^	*p*	IRR	95% CI	IRR		95% CI	*χ* ^2^	*p*
**Full cohort**													
**Number of children**					24.6	<.001						7.4	<.001
** No children**	1.58	(1.43–1.75)	1.86	(1.77–1.95)			1.29	(1.17–1.41)	1.58		(1.50–1.66)		<.01
** 1 child**	1.17	(1.08–1.26)	1.22	(1.16–1.28)			1.16	(1.09–1.24)	1.07		(1.01–1.14)		
** 2 children (Ref.)**	1.00		1.00				1.00		1.00				
** 3 children**	1.03	(0.95–1.11)	1.01	(0.96–1.07)			0.98	(0.92–1.06)	0.94		(0.87–1.01)		
** ≥4 children**	1.17	(1.06–1.30)	0.96	(0.89–1.04)		<.001	1.04	(0.94–1.15)	0.93		(0.85–1.03)		<.05
**All deceased**													
**Number of children**					18.5	<.01						11.3	<.05
** No children**	1.20	(1.05–1.37)	1.38	(1.29–1.48)			1.18	(1.05–1.32)	1.41		(1.32–1.50)		<.05
** 1 child**	1.05	(0.96–1.16)	1.06	(0.99–1.14)			1.10	(1.01–1.19)	1.04		(0.96–1.12)		
** 2 children (Ref.)**	1.00		1.00				1.00		1.00				
** 3 children**	0.98	(0.88–1.08)	0.97	(0.89–1.05)			0.99	(0.90–1.08)	0.92		(0.84–1.02)		
** ≥4 children**	1.13	(0.99–1.30)	0.84	(0.76–0.94)		<.00	0.98	(0.87–1.11)	0.92		(0.82–1.04)		
**Dementia deaths**													
**Number of children**					10.6	<.05						1.8	.766
** No children**	1.04	(0.90–1.20)	0.98	(0.90–1.06)			0.97	(0.83–1.15)	1.05		(0.95–1.17)		
** 1 child**	0.93	(0.82–1.04)	0.96	(0.88–1.04)			1.08	(0.96–1.20)	1.08		(0.96–1.22)		
** 2 children (Ref.)**	1.00		1.00				1.00		1.00				
** 3 children**	0.96	(0.86–1.07)	1.07	(0.98–1.16)			0.95	(0.84–1.07)	0.93		(0.81–1.06)		
** ≥4 children**	1.13	(0.97–1.32)	0.87	(0.76–1.00)		<.05	0.90	(0.77–1.06)	1.08		(0.92–1.27)		

*Note*. Models adjusted for age, age squared, region, calendar year, education level, and income.

## Geographic proximity

**Figure 2.**
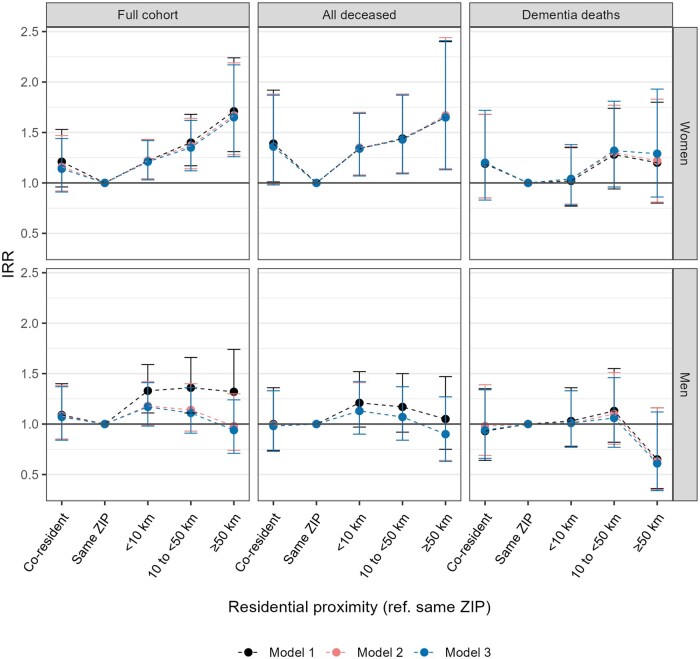
Incidence rate ratios with their 95% *CI* for annual care home days by residential proximity of the nearest child in the full cohort, among all deceased, and among those who died from dementia-related causes, Finnish women and men with at least one child and living in the Helsinki metropolitan area, 1998–2018. *Note*. Model 1 adjusted for age, age squared, calendar year, and the number of children; Model 2 adjusted for covariates in model 1 + marital status; Model 3 adjusted for covariates in model 2 + education level and income

Compared to living in the same ZIP code, a higher incidence of care home days was observed for women and men living <10 kilometers (IRR = 1.21, 95% CI: 1.04–1.43 for women; IRR = 1.33, 95% CI: 1.11–1.59 for men), 10 to <50 kilometers (IRR = 1.40, 95% CI: 1.17–1.68 for women; IRR = 1.36, 95% CI: 1.11–1.66 for men), and ≥50 km (IRR = 1.71, 95% CI: 1.31–2.24 for women; IRR = 1.32, 95% CI: 1.00–1.74 for men) from their nearest child in the full cohort of parents living in the Helsinki metropolitan area (Figure 2, model 1). Adjustments for marital status (model 2) attenuated the differences to a statistically nonsignificant level for men but had little impact on the estimates for women. The adjustment for socioeconomic status (model 3) had little further impact.

Among the deceased cohort members, the adjusted IRRs were generally similar to those of the full cohort in model 3. Among deceased women, co-residing with a child was associated with a higher incidence of care home days in model 1 (IRR = 1.39, 95% CI: 1.01–1.92), but this became statistically nonsignificant after adjusting for marital status in model 2 (IRR = 1.36, 95% CI: 0.99–1.88).

For women who died from dementia-related causes, IRRs for longer distances compared to living in the same ZIP code were smaller than those of all deceased or the full cohort, and were statistically nonsignificant in all adjustment models (<10 km: IRR = 1.04, 95% CI: 0.78–1.38; 10 to <50 km: IRR = 1.32, 95% CI: 0.96–1.81; ≥50 km: IRR = 1.29, 95% CI: 0.86–1.93; model 3). Among men, care home days did not vary by geographic proximity. All estimates are provided in Supplementary Table 3, see online supplementary material.

## Sensitivity and supplementary analyses

Logistic regression models for any care home use produced results consistent with the main Poisson analyses, indicating that the findings were not driven by the bimodal distribution of care home days (Supplementary Tables 4–6, see online supplementary material). Supplementary analysis showed that the presence of a daughter did not affect care home residence in any group (Supplementary Table 7, see online supplementary material). However, among deceased women, having a daughter—rather than a son—as the nearest child was associated with 17% more care home days (Supplementary Table 8, see online supplementary material).

## Discussion

This study investigated how care home residence varies by the number and geographic proximity of children among older adults in Finland, using register data with nearly complete fertility histories for both men and women. We found that the role of children differed for those who died from dementia-related causes compared to the general older population. Before dementia-related deaths, having children was not associated with care home residence, and any differences by geographic proximity were non-significant. In contrast, among all deceased, having at least one child was linked to fewer days in care homes, and closer proximity to children was associated with less care home residence among women. However, as overall care home use was low in this group, these differences, while statistically detectable, have little population-level impact. Overall, the findings suggest that children’s availability has a limited influence on care home use.

A unique contribution of our study is the separate analysis of individuals who died from dementia, compared with the general older population and those who died from any cause. This analytical approach allowed us to assess whether children reduce care home use among people with dementia—who are the primary users of these services—without bias from differing survival rates. Our results indicate that the typical pattern found in prior research does not extend to older adults with dementia. When care needs are highest, children’s ability to substitute for formal care in residential settings appears limited, as care home residence is likely driven more by disease progression than by the availability of family support (Korhonen et al., 2018). This diminished role of children may also reflect the broader caregiving context. In Finland—as in other Nordic countries—the state takes a central role in eldercare. Adult children are not legally obligated to provide or finance care, and there is generally a critical view toward filial responsibility (Kääriäinen et al., 2024). While informal care is common in the Nordic countries, it typically involves less high-intensity caregiving than in countries with less generous formal long-term care services (Verbakel, 2018). However, since this study focuses on Finland, comparative research is needed to determine whether the limited role of children in dementia-related care home use is specific to the Nordic welfare model.

Despite this, our findings confirm that having children is associated with less care home use in the general older population, both in the full cohort (unadjusted for health) and among the deceased (accounting for overall health status by design). This aligns with previous research showing that parents are less likely to enter care homes than those without children (Freedman, 1996; Grundy & Jitlal, 2007; Hedinger et al., 2015; Syse et al., 2022). However, unlike most earlier studies that rely on time-to-event analyses and focus on the timing of care home entry—potentially conflating differences in care use with differences in survival—our approach captures the volume of care home residence, offering a more comprehensive picture of the care burden before death.

A substantial share of the difference in care home residence between those with and without children appears to stem from the lower likelihood of partnership among the childless, while education and income contributed little to the observed gap. In addition to the absence of family caregiving, the association may partly reflect health-based selection into marriage and parenthood (Einiö et al., 2016; Fu & Goldman, 1996), which is not fully controlled for in our study design. Among men, children appeared particularly important in the absence of a spouse. As men tend to rely on their wives for informal care, adult children may take on a compensatory role following spousal loss, thereby reducing the need for care homes (Noël-Miller, 2010; van der Pers et al., 2015). In contrast to findings from the United States (Freedman, 1996; Himes et al., 2000), we found no evidence that the gender of the child influenced care home residence. Together with recent Swedish evidence (Artamonova et al., 2023), this may reflect a more gender-equal distribution of caregiving responsibilities and the predominantly instrumental nature of support provided.

Our findings also indicate that having more children does not necessarily lead to further reductions in care home residence. Prior studies have typically assumed a linear association, suggesting that each additional child lowers the likelihood of care home entry (Aykan, 2003; Coward et al., 1996; Cutler & Sheiner, 1994; Gaugler et al., 2007; Himes et al., 2000). Instead, our results suggest that even one child may provide sufficient support or take on greater caregiving responsibility to compensate for the lack of more adult children.

Shorter distances to children were associated with less care home residence in the full cohort and among the deceased, although co-residence did not further reduce care home use compared to living in the same ZIP code. Adjustment for marital status attenuated the association between child proximity and care home use among men, but had little effect among women, suggesting that proximity effects are largely independent of marital status for mothers, consistent with evidence that wives less commonly receive reciprocal care from their husbands (Katz et al., 2000). Similar gender differences were noted in Sweden, where living in the same neighborhood reduced the likelihood of care home entry more strongly for women than for men (Artamonova et al., 2023). Older women typically experience longer periods of disability at the end of life than men (Smith et al., 2013) and may, therefore, benefit more from having children nearby. Conversely, support for fathers might be less frequent or focus more on accessing formal home-care services, making children’s involvement less dependent on geographic proximity. These findings add to the body of research highlighting the benefits of having children nearby on parents’ well-being (van der Pers et al., 2015), stroke survival (Choi et al., 2022) and independent living (Artamonova et al., 2023; van der Pers et al., 2015), indicating that proximity to children can help women without dementia live at home longer.

## Methodological considerations

Utilizing register-based data enabled us to study a full cohort of older adults, integrating information from the care register with exact dates of care home entries and exits. Notably, the register linkage of children provides reliable data on fertility histories, which are often underreported in surveys, especially among men (Murphy, 2009; Rendall et al., 1999) and those suffering from dementia. Our analytic approach, which involved separate analysis for the deceased cohort members and those who died from dementia-related causes, effectively accounted for variations in health status and dementia-related care needs.

The study also has limitations. In Finland, the median age at care home entry was 81 years for men and 83 years for women in 1999, increasing to 84 and 86 years by 2018 (Korhonen et al., 2024). Because reliable linkage to children was available only for cohorts born since the late 1930s, who reached age 80 during the study period, the findings may not generalize to older ages. Prior studies suggest that children and spouses continue to reduce care home residence also beyond age 80 (Artamonova et al., 2023; Freedman, 1996; Martikainen et al., 2013), but whether the minimal role of children among those with dementia extends to higher ages remains uncertain. At more advanced ages, children may themselves have retired and be more available for care, yet the rising levels of frailty and multimorbidity with age could diminish the impact of informal caregiving. Future studies covering broader age ranges are, therefore, needed. Furthermore, our study focuses specifically on care home residence. While formal care services provided in-home may be important, especially for older adults with dementia, we were unable to assess this due to data limitations.

Because the analysis of geographic proximity was restricted to older adults living in the Helsinki metropolitan area, the results could differ for other regions of Finland, where residential patterns and care resources differ. More fine-grained data on place of residence would be needed to extend such analyses beyond the metropolitan area, as large ZIP code areas elsewhere currently limit precision.

Identifying dementia based on cause of death data may miss individuals who had dementia but died of other primary causes. To address this limitation, we adopted a multiple-cause approach by including cases in which dementia was recorded either as the underlying or a contributory cause of death, thereby increasing the sensitivity of our measure (Garcia-Ptacek et al., 2016). Finally, marital status is an imperfect proxy for caregiving availability, as some spouses may themselves be in care homes. Cohabiting partners can also be an important care resource, but because cohabitation status directly overlaps with the index person’s care home residence (individuals in care homes cannot cohabit), it risks capturing care home residence rather than caregiving resources. Moreover, previous research indicates that cohabiting unions are less protective against care home entry than marital unions (Moustgaard & Martikainen, 2009). We, therefore, consider marital status a more appropriate indicator.

## Conclusions

Having children is generally associated with reduced care home residence, with the presence of any children mattering more than their number. For men, children are particularly important in the absence of a spouse. For women, closer proximity is also associated with lower care home use. Crucially, however, these patterns do not extend to those with dementia—the group with the greatest care needs and the primary users of care homes. Before dementia-related deaths, having children was not associated with care home residence, and differences according to geographic proximity were minimal or absent. This suggests that while rising childlessness and the geographic dispersion of families may affect the availability of informal care, their impact on care home use is likely modest, given that dementia drives the bulk of care home use. Our findings, therefore, call into question the extent to which governments can reduce care home services by relying on families to fill the gap.

## Supplementary Material

gbaf250_Supplementary_Data

## Data Availability

This study was not preregistered. Access to data requires a signed access agreement with the register-holding institutions Statistics Finland and Health Data Permit Authority Findata after approval of a proposal.
