# Supplementary material for: The association of number and geographic proximity of children with care home use before all-cause and dementia deaths: a register-based study of Finnish older adults
Source: J Gerontol B Psychol Sci Soc Sci. 2025 Dec 10;81(2):gbaf250. doi: 10.1093/geronb/gbaf250 (PMC12795604; doi:10.1093/geronb/gbaf250)
Supplement: gbaf250_Supplementary_Data [file gbaf250_supplementary_data.zip › JGSS suppl Korhonen, Einiö, & Martikainen.docx]

***The Journals of Gerontology, Series B: Psychological Sciences and Social Sciences* Supplementary Material: Korhonen, Einiö, & Martikainen. The Association of Number and Geographic Proximity of Children and Care Home Use Before All-Cause and Dementia Deaths: A Register-Based Study of Finnish Older Adults.**

**
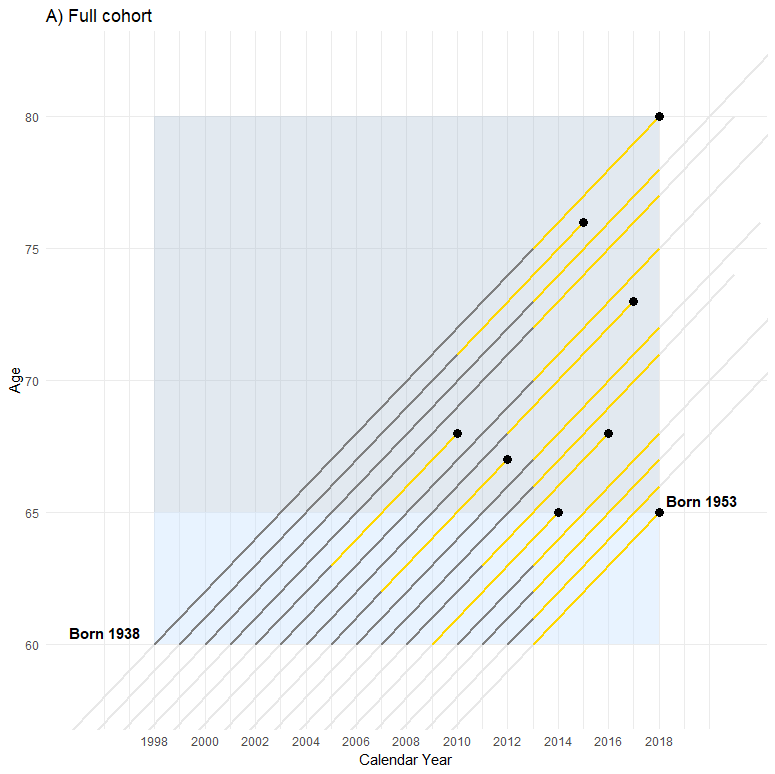

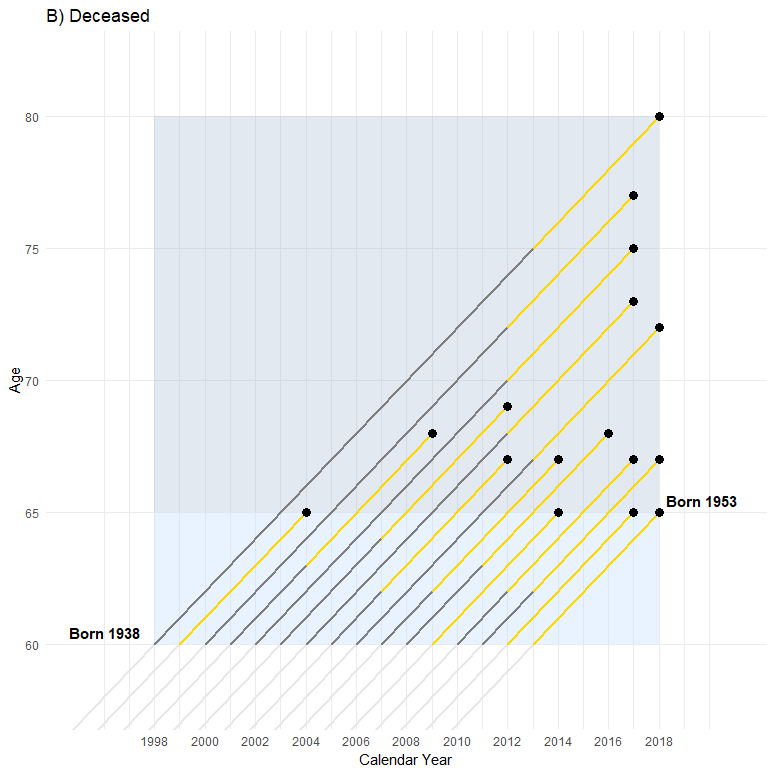
**

**Supplementary Figure S1.** Lexis diagrams illustrating the observation windows for care home residence for the full cohort (A) and deceased subsample (B).

Each person’s care home use was measured over a fixed five-year period. For survivors, this was the last five calendar years (2014–2018). For decedents, it was the five years before death at the age ≥65 years. Yellow lines show the observation period, and black dots mark deaths.

**Supplementary Table S1.** Distribution of annual care home days by gender in the full cohort, among all deceased, and among those who died from dementia-related causes, nationwide cohort of Finnish women and men, 1998–2018

|  | **Women** |  | **Men** |  |
| --- | --- | --- | --- | --- |
| Full cohort | Person-years | % | Person-years | % |
| Care home days |  |  |  |  |
| 0 | 2,668,235 | 97.8 | 2,499,225 | 97.6 |
| 1–364 | 23,977 | 0.9 | 27,417 | 1.1 |
| 365 | 36,853 | 1.4 | 35,213 | 1.4 |
| Total | 2,729,065 | 100.0 | 2,561,855 | 100.0 |
|  |  |  |  |  |
| All deceased | Person-years | % | Person-years | % |
| Care home days |  |  |  |  |
| 0 | 196,686 | 88.0 | 356,223 | 91.4 |
| 1–364 | 11,007 | 4.9 | 15,512 | 4.0 |
| 365 | 15,847 | 7.1 | 18,085 | 4.6 |
| Total | 223,540 | 100.0 | 389,820 | 100.0 |
|  |  |  |  |  |
| Dementia deaths | Person-years | % | Person-years | % |
| Care home days |  |  |  |  |
| 0 | 9874 | 50.5 | 13,162 | 62.1 |
| 1–364 | 2970 | 15.2 | 3157 | 14.9 |
| 365 | 6706 | 34.3 | 4886 | 23.0 |
| Total | 19,550 | 100.0 | 21,205 | 100.0 |

**Supplementary Table S2.** Incidence rate ratios (IRR) with their 95% confidence intervals (CI) for annual care home days by the number of children in the full cohort, among all deceased, and those who died from dementia-related causes, a nationwide cohort of Finnish women and men, 1998–2018

|  | **Women** | | | | | | **Men** | | | | | |
| --- | --- | --- | --- | --- | --- | --- | --- | --- | --- | --- | --- | --- |
|  | **Model 1** | | **Model 2** |  | **Model 3** | | **Model 1** | | **Model 2** |  | **Model 3** | |
| Full cohort | IRR | 95% CI | IRR | 95% CI | IRR | 95% CI | IRR | 95% CI | IRR | 95% CI | IRR | 95% CI |
| Number of children |  |  |  |  |  |  |  |  |  |  |  |  |
| No children | 2.34 | (2.24-2.44) | 1.62 | (1.54-1.71) | 1.66 | (1.58-1.75) | 2.89 | (2.78-3.01) | 1.59 | (1.50-1.67) | 1.48 | (1.41-1.56) |
| 1 child | 1.34 | (1.29-1.40) | 1.22 | (1.17-1.27) | 1.20 | (1.15-1.25) | 1.32 | (1.26-1.38) | 1.16 | (1.11-1.22) | 1.11 | (1.06-1.17) |
| 2 children (Ref.) | 1.00 |  | 1.00 |  | 1.00 |  | 1.00 |  | 1.00 |  | 1.00 |  |
| 3 children | 1.04 | (0.99-1.09) | 1.04 | (0.99-1.09) | 1.02 | (0.97-1.07) | 0.97 | (0.92-1.02) | 0.97 | (0.92-1.02) | 0.97 | (0.92-1.02) |
| ≥4 children | 1.12 | (1.05-1.19) | 1.08 | (1.01-1.15) | 1.03 | (0.97-1.10) | 1.04 | (0.97-1.11) | 1.00 | (0.94-1.08) | 0.98 | (0.91-1.05) |
|  |  |  |  |  |  |  |  |  |  |  |  |  |
| All deceased | IRR | 95% CI | IRR | 95% CI | IRR | 95% CI | IRR | 95% CI | IRR | 95% CI | IRR | 95% CI |
| Number of children |  |  |  |  |  |  |  |  |  |  |  |  |
| No children | 1.58 | (1.49-1.68) | 1.27 | (1.18-1.36) | 1.28 | (1.19-1.37) | 1.93 | (1.84-2.03) | 1.30 | (1.21-1.39) | 1.29 | (1.20-1.38) |
| 1 child | 1.12 | (1.05-1.18) | 1.06 | (1.00-1.12) | 1.06 | (1.00-1.12) | 1.15 | (1.08-1.21) | 1.07 | (1.01-1.13) | 1.06 | (1.00-1.12) |
| 2 children (Ref.) | 1.00 |  | 1.00 |  | 1.00 |  | 1.00 |  | 1.00 |  | 1.00 |  |
| 3 children | 0.98 | (0.92-1.05) | 0.98 | (0.92-1.04) | 0.97 | (0.91-1.04) | 0.96 | (0.90-1.03) | 0.96 | (0.90-1.02) | 0.96 | (0.90-1.03) |
| ≥4 children | 0.98 | (0.90-1.06) | 0.95 | (0.87-1.03) | 0.94 | (0.86-1.03) | 0.97 | (0.89-1.06) | 0.95 | (0.87-1.04) | 0.95 | (0.87-1.03) |
|  |  |  |  |  |  |  |  |  |  |  |  |  |
| Dementia deaths | IRR | 95% CI | IRR | 95% CI | IRR | 95% CI | IRR | 95% CI | IRR | 95% CI | IRR | 95% CI |
| Number of children |  |  |  |  |  |  |  |  |  |  |  |  |
| No children | 1.08 | (1.01-1.16) | 1.02 | (0.93-1.11) | 1.02 | (0.94-1.11) | 1.20 | (1.11-1.30) | 1.02 | (0.92-1.14) | 1.04 | (0.93-1.16) |
| 1 child | 0.97 | (0.91-1.04) | 0.95 | (0.89-1.02) | 0.96 | (0.89-1.02) | 1.11 | (1.02-1.20) | 1.08 | (1.00-1.17) | 1.08 | (1.00-1.17) |
| 2 children (Ref.) | 1.00 |  | 1.00 |  | 1.00 |  | 1.00 |  | 1.00 |  | 1.00 |  |
| 3 children | 1.02 | (0.95-1.09) | 1.01 | (0.94-1.08) | 1.02 | (0.95-1.09) | 0.95 | (0.87-1.04) | 0.95 | (0.87-1.04) | 0.95 | (0.87-1.04) |
| ≥4 children | 0.95 | (0.86-1.06) | 0.94 | (0.85-1.04) | 0.97 | (0.88-1.07) | 0.97 | (0.86-1.09) | 0.96 | (0.86-1.08) | 0.98 | (0.87-1.10) |

Model 1 adjusted for age, age squared, region, and calendar year; Model 2 adjusted for covariates in model 1 + marital status; Model 3 adjusted for covariates in model 2 + education level and income

**Supplementary Table S3.** Incidence rate ratios (IRR) with their 95% confidence intervals (CI) for annual care home days by the geographic proximity of the nearest child in the full cohort, among all deceased, and among those who died from dementia-related causes, women and men in living in the Helsinki metropolitan area, Finland 1998–2018

|  | **Women** | | | | | | **Men** | | | | | |
| --- | --- | --- | --- | --- | --- | --- | --- | --- | --- | --- | --- | --- |
|  | **Model 1** | | **Model 2** |  | **Model 3** | | **Model 1** | | **Model 2** |  | **Model 3** | |
| Full cohort | IRR | 95% CI | IRR | 95% CI | IRR | 95% CI | IRR | 95% CI | IRR | 95% CI | IRR | 95% CI |
| Geographic proximity |  |  |  |  |  |  |  |  |  |  |  |  |
| Co-resident | 1.21 | (0.96-1.53) | 1.16 | (0.92-1.47) | 1.14 | (0.91-1.44) | 1.09 | (0.85-1.40) | 1.08 | (0.85-1.38) | 1.07 | (0.84-1.37) |
| Same ZIP code (Ref.) | 1.00 |  | 1.00 |  | 1.00 |  | 1.00 |  | 1.00 |  | 1.00 |  |
| <10 km | 1.22 | (1.04-1.43) | 1.22 | (1.04-1.43) | 1.21 | (1.03-1.42) | 1.33 | (1.11-1.59) | 1.18 | (0.99-1.42) | 1.17 | (0.98-1.41) |
| 10 to < 50 km | 1.40 | (1.17-1.68) | 1.37 | (1.14-1.64) | 1.35 | (1.12-1.62) | 1.36 | (1.11-1.66) | 1.14 | (0.93-1.40) | 1.11 | (0.91-1.36) |
| ≥ 50 km | 1.71 | (1.31-2.24) | 1.67 | (1.28-2.19) | 1.65 | (1.26-2.17) | 1.32 | (1.00-1.74) | 0.98 | (0.74-1.30) | 0.94 | (0.71-1.24) |
| Unknown or child abroad | 1.18 | (0.80-1.75) | 1.20 | (0.81-1.78) | 1.24 | (0.84-1.85) | 1.18 | (0.70-2.00) | 0.95 | (0.57-1.59) | 0.91 | (0.54-1.53) |
|  |  |  |  |  |  |  |  |  |  |  |  |  |
| All deceased | IRR | 95% CI | IRR | 95% CI | IRR | 95% CI | IRR | 95% CI | IRR | 95% CI | IRR | 95% CI |
| Geographic proximity |  |  |  |  |  |  |  |  |  |  |  |  |
| Co-resident | 1.39 | (1.01-1.92) | 1.36 | (0.99-1.87) | 1.36 | (0.98-1.87) | 1.00 | (0.74-1.36) | 0.99 | (0.73-1.33) | 0.98 | (0.73-1.33) |
| Same ZIP code (Ref.) | 1.00 |  | 1.00 |  | 1.00 |  | 1.00 |  | 1.00 |  | 1.00 |  |
| <10 km | 1.34 | (1.07-1.69) | 1.35 | (1.08-1.70) | 1.34 | (1.07-1.69) | 1.21 | (0.97-1.52) | 1.13 | (0.90-1.41) | 1.13 | (0.90-1.42) |
| 10 to < 50 km | 1.44 | (1.10-1.88) | 1.43 | (1.10-1.88) | 1.43 | (1.09-1.87) | 1.17 | (0.92-1.50) | 1.07 | (0.84-1.37) | 1.07 | (0.84-1.37) |
| ≥ 50 km | 1.65 | (1.14-2.40) | 1.67 | (1.14-2.44) | 1.65 | (1.13-2.41) | 1.05 | (0.75-1.47) | 0.90 | (0.64-1.27) | 0.90 | (0.63-1.27) |
| Unknown or child abroad | 1.36 | (0.75-2.45) | 1.38 | (0.77-2.49) | 1.36 | (0.75-2.47) | 0.94 | (0.51-1.73) | 0.90 | (0.50-1.62) | 0.85 | (0.45-1.57) |
|  |  |  |  |  |  |  |  |  |  |  |  |  |
| Dementia death | IRR | 95% CI | IRR | 95% CI | IRR | 95% CI | IRR | 95% CI | IRR | 95% CI | IRR | 95% CI |
| Geographic proximity |  |  |  |  |  |  |  |  |  |  |  |  |
| Co-resident | 1.19 | (0.85-1.68) | 1.20 | (0.85-1.68) | 1.20 | (0.83-1.72) | 0.93 | (0.64-1.35) | 0.98 | (0.69-1.39) | 0.94 | (0.66-1.34) |
| Same ZIP code (Ref.) | 1.00 |  | 1.00 |  | 1.00 |  | 1.00 |  | 1.00 |  | 1.00 |  |
| <10 km | 1.02 | (0.77-1.35) | 1.04 | (0.79-1.36) | 1.04 | (0.78-1.38) | 1.03 | (0.78-1.36) | 1.01 | (0.77-1.33) | 1.01 | (0.77-1.33) |
| 10 to < 50 km | 1.28 | (0.94-1.74) | 1.30 | (0.96-1.77) | 1.32 | (0.96-1.81) | 1.13 | (0.82-1.55) | 1.10 | (0.80-1.51) | 1.06 | (0.77-1.46) |
| ≥ 50 km | 1.20 | (0.80-1.80) | 1.22 | (0.81-1.83) | 1.29 | (0.86-1.93) | 0.65 | (0.36-1.16) | 0.63 | (0.35-1.16) | 0.61 | (0.34-1.12) |
| Unknown or child abroad | 0.73 | (0.36-1.50) | 0.74 | (0.37-1.47) | 0.70 | (0.36-1.34) | 0.56 | (0.20-1.52) | 0.64 | (0.28-1.45) | 0.51 | (0.18-1.47) |

Model 1 adjusted for age, age squared, number of children, and calendar year; Model 2 adjusted for covariates in model 1 + marital status; Model 3 adjusted for covariates in model 2 + education level and income

**Supplementary Table S4.** Odds ratios (OR) with their 95% confidence intervals for any care home use across each year by the number of children in the full cohort, among all deceased, and those who died from dementia-related causes, a nationwide cohort of Finnish women and men, 1998–2018

|  | **Women** | | | | | | **Men** | | | | | |
| --- | --- | --- | --- | --- | --- | --- | --- | --- | --- | --- | --- | --- |
|  | **Model 1** | | **Model 2** |  | **Model 3** | | **Model 1** | | **Model 2** |  | **Model 3** | |
| Full cohort | OR | 95% CI | OR | 95% CI | OR | 95% CI | OR | 95% CI | OR | 95% CI | OR | 95% CI |
| Number of children |  |  |  |  |  |  |  |  |  |  |  |  |
| No children | 2.36 | (2.27-2.46) | 1.65 | (1.56-1.73) | 1.68 | (1.60-1.77) | 2.83 | (2.73-2.94) | 1.57 | (1.49-1.64) | 1.46 | (1.39-1.54) |
| 1 child | 1.34 | (1.29-1.40) | 1.22 | (1.17-1.27) | 1.20 | (1.15-1.25) | 1.31 | (1.25-1.36) | 1.16 | (1.11-1.21) | 1.11 | (1.06-1.16) |
| 2 children (Ref.) | 1.00 |  | 1.00 |  | 1.00 |  | 1.00 |  | 1.00 |  | 1.00 |  |
| 3 children | 1.03 | (0.98-1.08) | 1.03 | (0.98-1.07) | 1.01 | (0.96-1.05) | 0.99 | (0.94-1.03) | 0.99 | (0.94-1.03) | 0.98 | (0.94-1.03) |
| ≥4 children | 1.15 | (1.08-1.22) | 1.11 | (1.05-1.18) | 1.05 | (0.99-1.12) | 1.04 | (0.98-1.11) | 1.01 | (0.95-1.08) | 0.98 | (0.92-1.04) |
|  |  |  |  |  |  |  |  |  |  |  |  |  |
| All deceased | OR | 95% CI | OR | 95% CI | OR | 95% CI | OR | 95% CI | OR | 95% CI | OR | 95% CI |
| Number of children |  |  |  |  |  |  |  |  |  |  |  |  |
| No children | 1.66 | (1.56-1.76) | 1.31 | (1.21-1.41) | 1.32 | (1.22-1.42) | 1.95 | (1.86-2.05) | 1.31 | (1.23-1.41) | 1.30 | (1.21-1.39) |
| 1 child | 1.13 | (1.06-1.20) | 1.07 | (1.00-1.13) | 1.06 | (1.00-1.13) | 1.14 | (1.08-1.21) | 1.07 | (1.01-1.13) | 1.06 | (1.00-1.12) |
| 2 children (Ref.) | 1.00 |  | 1.00 |  | 1.00 |  | 1.00 |  | 1.00 |  | 1.00 |  |
| 3 children | 0.97 | (0.91-1.04) | 0.97 | (0.90-1.03) | 0.96 | (0.89-1.03) | 0.98 | (0.92-1.04) | 0.97 | (0.91-1.04) | 0.97 | (0.91-1.04) |
| ≥4 children | 0.99 | (0.90-1.08) | 0.95 | (0.87-1.04) | 0.94 | (0.86-1.03) | 0.98 | (0.90-1.06) | 0.96 | (0.88-1.04) | 0.96 | (0.88-1.04) |
|  |  |  |  |  |  |  |  |  |  |  |  |  |
| Dementia deaths | OR | 95% CI | OR | 95% CI | OR | 95% CI | OR | 95% CI | OR | 95% CI | OR | 95% CI |
| Number of children |  |  |  |  |  |  |  |  |  |  |  |  |
| No children | 1.17 | (1.02-1.33) | 1.06 | (0.91-1.24) | 1.07 | (0.91-1.25) | 1.28 | (1.13-1.44) | 0.99 | (0.84-1.17) | 1.02 | (0.86-1.20) |
| 1 child | 0.95 | (0.84-1.08) | 0.91 | (0.80-1.03) | 0.92 | (0.81-1.04) | 1.14 | (1.01-1.29) | 1.10 | (0.98-1.25) | 1.11 | (0.98-1.25) |
| 2 children (Ref.) | 1.00 |  | 1.00 |  | 1.00 |  | 1.00 |  | 1.00 |  | 1.00 |  |
| 3 children | 1.02 | (0.89-1.16) | 1.00 | (0.88-1.14) | 1.02 | (0.90-1.17) | 0.93 | (0.82-1.06) | 0.93 | (0.82-1.06) | 0.93 | (0.82-1.06) |
| ≥4 children | 0.95 | (0.8-1.13) | 0.92 | (0.76-1.10) | 0.97 | (0.81-1.16) | 1.00 | (0.85-1.18) | 0.99 | (0.84-1.16) | 1.01 | (0.86-1.19) |

Model 1 adjusted for age, age squared, region, and calendar year; Model 2 adjusted for covariates in model 1 + marital status; Model 3 adjusted for covariates in model 2 + education level and income

**Supplementary Table S5.** Odds ratios (OR) with their 95% confidence intervals (CI) for any care home use across each year by the number of children and marital status, a nationwide sample of Finnish women and men, 1998–2018

|  | **Women** | | | |  | **Men** | | | |  |
| --- | --- | --- | --- | --- | --- | --- | --- | --- | --- | --- |
|  | **Married** | | **Non-married** | |  | **Married** | | **Non-married** | |  |
| Full cohort | OR | 95% CI | OR | 95% CI | Test for interaction | OR | 95% CI | OR | 95% CI | Test for interaction |
| Number of children |  |  |  |  | *Wald χ2(4)=25.6, P<0.001* |  |  |  |  | *Wald χ2(4)=36.5, P<0.001* |
| No children | 1.60 | (1.46-1.75) | 1.88 | (1.79-1.97) | p<0.01 | 1.30 | (1.19-1.41) | 1.55 | (1.48-1.62) | p<0.001 |
| 1 child | 1.18 | (1.11-1.27) | 1.22 | (1.16-1.28) |  | 1.16 | (1.09-1.23) | 1.06 | (1.00-1.12) |  |
| 2 children (Ref.) | 1.00 |  | 1.00 |  |  | 1.00 |  | 1.00 |  |  |
| 3 children | 1.02 | (0.95-1.10) | 0.99 | (0.94-1.05) |  | 1.02 | (0.95-1.08) | 0.94 | (0.88-1.01) |  |
| ≥4 children | 1.17 | (1.06-1.28) | 0.99 | (0.91-1.06) | p<0.001 | 1.03 | (0.95-1.12) | 0.93 | (0.84-1.01) | <0.05 |
|  |  |  |  |  |  |  |  |  |  |  |
| All deceased | OR | 95% CI | OR | 95% CI | Test for interaction | OR | 95% CI | OR | 95% CI | Test for interaction |
| Number of children |  |  |  |  | *Wald χ2(4)=19.1, P<0.001* |  |  |  |  | *Wald χ2(4)=16.7, P<0.01* |
| No children | 1.23 | (1.08-1.40) | 1.45 | (1.34-1.56) |  | 1.19 | (1.07-1.33) | 1.42 | (1.32-1.51) | p<0.01 |
| 1 child | 1.07 | (0.98-1.18) | 1.07 | (0.98-1.15) |  | 1.10 | (1.02-1.20) | 1.02 | (0.94-1.11) |  |
| 2 children (Ref.) | 1.00 |  | 1.00 |  |  | 1.00 |  | 1.00 |  |  |
| 3 children | 0.97 | (0.88-1.07) | 0.95 | (0.87-1.05) |  | 1.01 | (0.93-1.09) | 0.93 | (0.84-1.02) |  |
| ≥4 children | 1.13 | (0.99-1.29) | 0.84 | (0.75-0.95) | p<0.01 | 0.99 | (0.89-1.11) | 0.91 | (0.81-1.03) |  |
|  |  |  |  |  |  |  |  |  |  |  |
| Dementia deaths | OR | 95% CI | OR | 95% CI | Test for interaction | OR | 95% CI | OR | 95% CI | Test for interaction |
| Number of children |  |  |  |  | *Wald χ2(4)=13.0, P<0.05* |  |  |  |  | *Wald χ2(4)=4.7, P=0.315* |
| No children | 1.06 | (0.84-1.35) | 0.98 | (0.82-1.16) |  | 0.94 | (0.76-1.16) | 1.04 | (0.87-1.24) |  |
| 1 child | 0.90 | (0.75-1.07) | 0.92 | (0.77-1.11) |  | 1.10 | (0.95-1.28) | 1.12 | (0.90-1.38) |  |
| 2 children (Ref.) | 1.00 |  | 1.00 |  |  | 1.00 |  | 1.00 |  |  |
| 3 children | 0.92 | (0.77-1.10) | 1.15 | (0.94-1.41) |  | 0.94 | (0.81-1.10) | 0.86 | (0.68-1.09) |  |
| ≥4 children | 1.22 | (0.95-1.57) | 0.79 | (0.61-1.01) | p<0.01 | 0.90 | (0.73-1.10) | 1.27 | (0.96-1.67) |  |

Models adjusted for age, age squared, region, calendar year, education level, and income

**Supplementary Table S6.** Odds ratios (OR) with their 95% confidence intervals (CI) for any care home use across each year by the geographic proximity of the nearest child in the full cohort, among all deceased, and among those who died from dementia-related causes, women and men living in the Helsinki metropolitan area, Finland 1998–2018

|  | **Women** | | | | | | **Men** | | | | | |
| --- | --- | --- | --- | --- | --- | --- | --- | --- | --- | --- | --- | --- |
|  | **Model 1** | | **Model 2** | | **Model 3** | | **Model 1** | | **Model 2** | | **Model 3** | |
| Full cohort | OR | 95% CI | OR | 95% CI | OR | 95% CI | OR | 95% CI | OR | 95% CI | OR | 95% CI |
| Geographic proximity |  |  |  |  |  |  |  |  |  |  |  |  |
| Co-resident | 1.22 | (0.99-1.51) | 1.17 | (0.95-1.45) | 1.15 | (0.93-1.42) | 1.08 | (0.87-1.34) | 1.07 | (0.86-1.33) | 1.06 | (0.86-1.32) |
| Same ZIP code (Ref.) | 1.00 |  | 1.00 |  | 1.00 |  | 1.00 |  | 1.00 |  | 1.00 |  |
| <10 km | 1.26 | (1.09-1.45) | 1.24 | (1.07-1.43) | 1.24 | (1.07-1.43) | 1.30 | (1.11-1.52) | 1.16 | (0.99-1.36) | 1.15 | (0.98-1.35) |
| 10 to < 50 km | 1.36 | (1.15-1.61) | 1.33 | (1.13-1.57) | 1.31 | (1.11-1.55) | 1.34 | (1.12-1.60) | 1.12 | (0.94-1.34) | 1.09 | (0.91-1.31) |
| ≥ 50 km | 1.70 | (1.32-2.18) | 1.61 | (1.25-2.07) | 1.59 | (1.23-2.05) | 1.40 | (1.09-1.78) | 1.04 | (0.81-1.33) | 0.99 | (0.77-1.26) |
| Unknown or child abroad | 1.25 | (0.87-1.79) | 1.24 | (0.86-1.79) | 1.28 | (0.89-1.84) | 1.13 | (0.72-1.78) | 0.92 | (0.58-1.45) | 0.90 | (0.57-1.42) |
|  |  |  |  |  |  |  |  |  |  |  |  |  |
| All deceased | OR | 95% CI | OR | 95% CI | OR | 95% CI | OR | 95% CI | OR | 95% CI | OR | 95% CI |
| Geographic proximity |  |  |  |  |  |  |  |  |  |  |  |  |
| Co-resident | 1.43 | (1.05-1.95) | 1.40 | (1.03-1.91) | 1.40 | (1.02-1.91) | 1.06 | (0.80-1.39) | 1.05 | (0.80-1.38) | 1.04 | (0.79-1.37) |
| Same ZIP code (Ref.) | 1.00 |  | 1.00 |  | 1.00 |  | 1.00 |  | 1.00 |  | 1.00 |  |
| <10 km | 1.40 | (1.13-1.75) | 1.41 | (1.13-1.75) | 1.40 | (1.13-1.75) | 1.20 | (0.97-1.47) | 1.13 | (0.92-1.39) | 1.12 | (0.91-1.39) |
| 10 to < 50 km | 1.40 | (1.08-1.81) | 1.40 | (1.08-1.82) | 1.40 | (1.08-1.81) | 1.20 | (0.95-1.50) | 1.10 | (0.87-1.39) | 1.10 | (0.87-1.38) |
| ≥ 50 km | 1.63 | (1.11-2.39) | 1.63 | (1.11-2.39) | 1.61 | (1.09-2.36) | 1.20 | (0.88-1.63) | 1.03 | (0.75-1.42) | 1.02 | (0.75-1.40) |
| Unknown or child abroad | 1.53 | (0.86-2.72) | 1.55 | (0.88-2.76) | 1.53 | (0.86-2.73) | 0.95 | (0.55-1.62) | 0.86 | (0.50-1.48) | 0.86 | (0.5-1.49) |
|  |  |  |  |  |  |  |  |  |  |  |  |  |
| Dementia deaths | OR | 95% CI | OR | 95% CI | OR | 95% CI | OR | 95% CI | OR | 95% CI | OR | 95% CI |
| Geographic proximity |  |  |  |  |  |  |  |  |  |  |  |  |
| Co-resident | 1.36 | (0.79-2.34) | 1.38 | (0.79-2.38) | 1.45 | (0.81-2.57) | 0.94 | (0.57-1.56) | 0.98 | (0.59-1.62) | 0.91 | (0.55-1.51) |
| Same ZIP code (Ref.) | 1.00 |  | 1.00 |  | 1.00 |  | 1.00 |  | 1.00 |  | 1.00 |  |
| <10 km | 0.98 | (0.66-1.47) | 1.01 | (0.67-1.52) | 1.04 | (0.68-1.57) | 0.98 | (0.67-1.44) | 0.97 | (0.66-1.43) | 0.95 | (0.65-1.40) |
| 10 to < 50 km | 1.41 | (0.88-2.27) | 1.46 | (0.90-2.36) | 1.54 | (0.94-2.52) | 1.14 | (0.71-1.82) | 1.12 | (0.69-1.80) | 1.06 | (0.66-1.71) |
| ≥ 50 km | 1.17 | (0.6-2.30) | 1.23 | (0.62-2.43) | 1.32 | (0.67-2.60) | 0.86 | (0.44-1.71) | 0.87 | (0.43-1.76) | 0.83 | (0.42-1.66) |
| Unknown or child abroad | 0.61 | (0.23-1.6) | 0.60 | (0.24-1.50) | 0.57 | (0.24-1.36) | 0.49 | (0.18-1.29) | 0.49 | (0.18-1.31) | 0.45 | (0.16-1.31) |

Model 1 adjusted for age, age squared, number of children, and calendar year; Model 2 adjusted for covariates in model 1 + marital status; Model 3 adjusted for covariates in model 2 + education level and income

**Supplementary Table S7.** Having at least one daughter and annual care home days, a nationwide sample of Finnish women and men, 1998–2018. Incidence rate ratios (IRR) with their 95% confidence intervals (CI), adjusted for age, age squared, region, calendar year, number of children, marital status, education level, and income

|  | Women | | Men | |
| --- | --- | --- | --- | --- |
| Full cohort | IRR | 95% CI | IRR | 95% CI |
| At least one daughter |  |  |  |  |
| No (Ref.) | 1.00 |  | 1.00 |  |
| Yes | 0.98 | (0.94-1.02) | 0.97 | (0.92-1.01) |
|  |  |  |  |  |
| All deceased | IRR | 95% CI | IRR | 95% CI |
| At least one daughter |  |  |  |  |
| No (Ref.) | 1.00 |  | 1.00 |  |
| Yes | 0.96 | (0.91-1.02) | 0.97 | (0.92-1.03) |
|  |  |  |  |  |
| Dementia deaths | IRR | 95% CI | IRR | 95% CI |
| At least one daughter |  |  |  |  |
| No (Ref.) | 1.00 |  | 1.00 |  |
| Yes | 1.00 | (1.00-1.00) | 0.99 | (0.92-1.07) |

**Supplementary Table S8.** Gender of the nearest living child and annual care home days, women and men living in the Helsinki metropolitan area, Finland 1998–2018. Incidence rate ratios (IRR) with their 95% confidence intervals (CI), adjusted for age, age squared, calendar year, number of children, marital status, education level, and income

|  | **Women** |  | **Men** |  |
| --- | --- | --- | --- | --- |
| Full cohort | IRR | 95% CI | IRR | 95% CI |
| Gender of nearest child |  |  |  |  |
| Son (Ref.) | 1.00 |  | 1.00 |  |
| Daughter | 1.02 | (0.93-1.12) | 0.95 | (0.87-1.04) |
|  |  |  |  |  |
| All deceased | IRR | 95% CI | IRR | 95% CI |
| Gender of nearest child |  |  |  |  |
| Son (Ref.) | 1.00 |  | 1.00 |  |
| Daughter | 1.17 | (1.02-1.33) | 1.01 | (0.90-1.13) |
|  |  |  |  |  |
| Dementia deaths | IRR | 95% CI | IRR | 95% CI |
| Gender of nearest child |  |  |  |  |
| Son (Ref.) | 1.00 |  | 1.00 |  |
| Daughter | 1.08 | (0.91-1.28) | 1.14 | (0.98-1.32) |
